# Remineralization Potential of Self-Assembling Peptides Versus Fluoride Agents in White Spot Lesions: A Systematic Review

**DOI:** 10.7759/cureus.95416

**Published:** 2025-10-25

**Authors:** Babitha Babu, Dimple Padawe, Vilas Takate

**Affiliations:** 1 Pedodontics and Preventive Dentistry, Government Dental College and Hospital, Mumbai, Mumbai, IND

**Keywords:** enamel remineralization, fluoride varnish, self-assembling peptides, systematic review, white spot lesions

## Abstract

White spot lesions (WSLs) are early manifestations of enamel demineralization, commonly observed in orthodontic patients. Traditional fluoride therapies promote surface-level remineralization but often fall short in addressing subsurface mineral loss. Self-assembling peptides (SAPs), particularly P11-4, have emerged as biomimetic agents capable of penetrating lesions and facilitating deeper enamel regeneration. This systematic review aims to compare the remineralization potential of SAPs and fluoride agents in managing WSLs, evaluating both clinical efficacy and underlying mechanisms. Following Preferred Reporting Items for Systematic Reviews and Meta-Analyses (PRISMA) guidelines and registered in PROSPERO (CRD42024622963), an extensive literature search was conducted across PubMed, MEDLINE, Embase, Cochrane, Scopus, and grey literature from January 2000 to December 2024, which yielded 1,028 potential records. After screening 665 titles, 180 abstracts, and 60 retrieved full texts, seven clinical studies, all randomized or controlled clinical trials, met the eligibility criteria. Each study was assessed for risk of bias using the Cochrane RoB 2 tool, and evidence quality was evaluated using the Grading of Recommendations, Assessment, Development, and Evaluation (GRADE) approach. A meta-analysis was not performed due to methodological heterogeneity; therefore, results were synthesized narratively. SAP P11-4 demonstrated superior subsurface remineralization compared to fluoride in all included studies, with DIAGNOdent measurements showing reductions ranging from 23% to 41%. Combined SAP-fluoride therapy yielded enhanced outcomes. Although visual changes (International Caries Detection and Assessment System (ICDAS) scores) were often indistinguishable between groups, significant differences in mineral recovery were consistently reported. SAP P11-4 is a promising, minimally invasive alternative to fluoride for managing WSLs, especially in orthodontic patients. Its capacity for deep remineralization highlights its potential as a next-generation caries management tool. However, findings are based on short-term clinical trials, and further long-term randomized studies are recommended to validate and optimize clinical protocols.

## Introduction and background

White spot lesions (WSLs) are among the earliest clinical signs of enamel demineralization, appearing as opaque white areas on smooth surfaces of teeth while maintaining an intact outer enamel layer [1]. These subsurface lesions are primarily associated with an acidic oral environment, often exacerbated by poor plaque control around orthodontic appliances, along gingival margins, and in stagnant areas of the dentition [2]. WSLs represent a critical early stage in the caries continuum, reflecting a disruption in the equilibrium between demineralization and remineralization [3]. Without timely intervention, these lesions may progress to cavitation, compromising both dental function and aesthetics [4].

Fluoride-based therapies have long been the mainstay in managing WSLs, owing to their well-documented ability to promote remineralization and suppress further demineralization [5]. Fluoride forms fluorapatite which is a mineral more acid-resistant than hydroxyapatite, and thereby enhances enamel resistance to acidic challenges [6]. Additionally, fluoride inhibits bacterial activity and supports the redeposition of calcium and phosphate ions into the enamel. However, despite these advantages, fluoride-induced remineralization tends to be surface-limited, often failing to penetrate and restore the deeper mineral loss within the lesion body [7,8]. This surface-centric effect has prompted the development of adjunctive or alternative remineralizing agents capable of targeting the subsurface lesion.

Among such innovations, self-assembling peptides (SAPs) have emerged as a biomimetic approach that closely mimics natural enamel regeneration processes [9]. SAPs, such as the P11-4 peptide, self-assemble into three-dimensional scaffolds upon exposure to ionic fluids in the oral cavity, guiding hydroxyapatite nucleation within the lesion [10]. These scaffolds offer a framework that facilitates deep mineral infiltration and structural restoration of enamel. Unlike fluoride, which predominantly induces mineral deposition on the outer enamel surface forming a fluorapatite-rich layer, SAP P11-4 penetrates into the lesion body, assembling into a fibrous matrix that promotes hydroxyapatite crystal nucleation from within. This allows progressive subsurface remineralization and structural recovery of the deeper layers of enamel [10,11]. Importantly, SAPs exhibit lesion-specific activity, targeting demineralized enamel while preserving unaffected areas [11].

Direct comparisons between SAPs and fluoride agents provide critical insights into their relative effectiveness and practical utility in clinical scenarios. While fluoride remains an integral part of public health caries prevention strategies, SAPs may offer distinct advantages in situations requiring focused and deeper remineralization [12-14]. Evidence suggests that SAPs can induce more homogenous mineral deposition and may be less dependent on frequent application or patient compliance [15,16].

The clinical relevance of these advances is particularly pronounced in orthodontic populations, where WSLs are a frequent complication due to prolonged appliance wear and associated plaque accumulation [17]. In such cases, the limitations of fluoride may necessitate alternative interventions such as SAPs to ensure effective, minimally invasive treatment. The growing emphasis on preventive and conservative dentistry further underscores the need for therapies that restore enamel integrity without resorting to invasive procedures [18].

Despite growing evidence supporting both fluoride and SAPs individually, existing literature lacks comprehensive systematic reviews that directly compare their clinical efficacy using standardized quantitative measures. This knowledge gap underscores the need for evidence synthesis to establish their relative effectiveness and guide clinical decision-making. In this context, the present systematic review aims to critically compare the remineralization efficacy of SAPs and fluoride agents in WSLs, evaluating their mechanisms, outcomes, and clinical utility. Such an appraisal may offer meaningful contributions toward advancing non-invasive caries management and reinforcing the role of biomimetic strategies in restorative and preventive care.

## Review

Methodology

The primary objective of this systematic review was to address the research question: "What is the comparative remineralization potential of SAPs versus fluoride agents in managing WSLs?" The review adhered to the Preferred Reporting Items for Systematic Reviews and Meta-Analyses (PRISMA) guidelines [19], and was registered in the International Prospective Register of Systematic Reviews (PROSPERO) database (Ref ID: CRD42024622963).

Search Strategy

The literature search was performed systematically across multiple databases to identify all relevant studies addressing the research question. The databases searched included PubMed, MEDLINE, Embase, the Cochrane Library, Scopus, and Google Scholar. Additional searches were conducted using clinical trial registries such as ClinicalTrials.gov and grey literature sources, including conference proceedings, dissertations, and preprint archives, to capture unpublished or non-indexed studies.

The search strategy was developed in consultation with a librarian specializing in systematic reviews to ensure accuracy and comprehensiveness. A combination of Medical Subject Headings (MeSH) terms and free-text keywords was employed. These terms included “SAP,” “fluoride agents,” “white spot lesions,” “remineralization,” “dental enamel,” “enamel demineralization,” and “lesion management.” Boolean operators such as “AND,” “OR,” and “NOT” were used to refine search results and ensure precision. Database-specific filters were applied to limit the results to studies published in English between January 2000 and December 2024. This timeframe was selected to focus on contemporary advancements in remineralization technologies. A sample search string for PubMed is provided in the Appendix. Duplicates were removed using reference management software to streamline the screening process. 

Eligibility Criteria

The Population, Intervention, Comparator, Outcomes, Study Design (PICOS) framework used to determine the eligibility of the studies in the present systematic review is listed in Table 1.

**Table 1:** Population, Intervention, Comparator, Outcomes, Study Design (PICOS) Criteria with Inclusion and Exclusion Criteria

PICOS Component	Inclusion Criteria	Exclusion Criteria
Population	Individuals of all ages with clinically or radiographically diagnosed white spot lesions (WSLs). Individuals with WSLs resulting from orthodontic treatment or naturally occurring lesions.	Individuals with systemic conditions affecting enamel structure (e.g., amelogenesis imperfecta). Patients with cavitated lesions or severe caries requiring restorative intervention.
Intervention	Studies evaluating self-assembling peptides (SAPs) explicitly designed for enamel remineralization (e.g., P11-4). Studies reporting clinical, radiographic, or biochemical outcomes related to remineralization.	Studies using SAPs in animal models or in vitro studies. SAPs used for purposes other than enamel remineralization (e.g., bone regeneration).
Comparator	Studies involving fluoride-based agents, including gels, mouth rinses, or toothpaste. Studies directly comparing SAPs with fluoride-based agents.	Studies without an active comparator. Studies combining fluoride with other remineralizing agents.
Outcomes	Primary: Remineralization assessed by quantitative light-induced fluorescence (QLF), microradiography, or clinical/radiographic measures of lesion depth reduction. Secondary: Aesthetic improvements evaluated through patient-reported outcomes or visual scales. Adverse effects, including sensitivity or allergic reactions. Patient compliance and long-term stability of remineralized lesions.	Studies not reporting quantitative remineralization outcomes. Outcomes unrelated to enamel health.
Study Design	Randomized controlled trials (RCTs). Controlled clinical trials (CCTs). Prospective cohort studies.	Animal studies, in vitro studies, case reports, reviews, and studies with insufficient data on outcomes.

Population

The population of interest for this review included individuals of all age groups with clinically or radiographically diagnosed white spot lesions. Participants with WSLs resulting from orthodontic treatment or naturally occurring lesions unrelated to braces were eligible for inclusion. This broad inclusion criterion ensured that the review captured a diverse range of clinical scenarios, enhancing the generalizability of the findings. Exclusion criteria were carefully defined to avoid confounding factors that could impact the interpretation of results. Individuals with systemic conditions affecting enamel structure, such as amelogenesis imperfecta, were excluded to maintain a focus on typical cases of enamel demineralization. Furthermore, patients with cavitated lesions or severe caries requiring restorative intervention were excluded to ensure that the review concentrated on non-invasive remineralization strategies applicable to early-stage lesions.

Interventions

The intervention of interest in this review was the use of SAP for the remineralization of white spot lesions. SAP formulations, such as P11-4, designed explicitly for enamel repair, were included. These peptides are biomimetic agents that form a three-dimensional scaffold within demineralized lesions, promoting the nucleation and deposition of hydroxyapatite crystals [10]. Studies evaluating SAPs in clinical, radiographic, or biochemical contexts were included in the review. However, studies investigating SAPs in animal models, in vitro settings, or for purposes unrelated to enamel remineralization were excluded.

Comparators

The comparator group comprised fluoride-based agents, which are widely recognized as the standard of care for the management of white spot lesions. Fluoride agents included in the review consisted of topical fluoride gels, mouth rinses, toothpaste, and other formulations designed for enamel remineralization. Studies that directly compared the efficacy of SAPs to fluoride-based agents were eligible for inclusion. Studies without an active comparator or those combining fluoride with other remineralizing agents were excluded, as these could confound the interpretation of the findings.

Outcomes

The review focused on both primary and secondary outcomes to provide a comprehensive evaluation of the interventions. The primary outcomes included the degree of remineralization as measured by quantitative light-induced fluorescence (QLF), microradiographic analysis of mineral gain, and reductions in lesion depth or size assessed clinically or radiographically. These outcomes were chosen to quantitatively assess the effectiveness of the interventions. Secondary outcomes included aesthetic improvements evaluated through patient-reported measures or standardized visual scales, adverse effects such as tooth sensitivity or allergic reactions, compliance with the intervention protocol, and the long-term stability of remineralized lesions. These secondary outcomes aimed to capture patient-centered and practical considerations associated with the interventions.

Study Selection

The study selection process was conducted in two stages. Initially, titles and abstracts retrieved from the search were screened independently by two reviewers to identify studies that met the inclusion criteria. This step involved applying the predefined eligibility criteria to each record to exclude irrelevant studies. In cases where the relevance of a study was unclear based on the title and abstract, the full text was retrieved for further assessment. The second stage involved a detailed review of the full texts of all potentially eligible studies. Each study was evaluated independently by two reviewers, with any discrepancies resolved through discussion or consultation with a third reviewer. The entire study selection process was documented using a PRISMA flow diagram, which detailed the number of records identified, screened, excluded, and included at each stage.

Data Extraction and Synthesis

Data extraction was performed independently by two reviewers using a standardized data extraction form. The extracted data included study characteristics (author, year, country, and study design), population details (sample size, age, and sex distribution), intervention characteristics (type of SAP, concentration, and application protocol), comparator characteristics (type of fluoride agent, concentration, and application protocol), and outcomes (lesion depth reduction, aesthetic improvement, patient compliance). Additional details, such as the duration of follow-up and risk of bias assessments, were also recorded. The extracted data were cross-verified by the reviewers to ensure accuracy and completeness. Any disagreements were resolved through discussion and consensus. A narrative synthesis of the included studies was conducted, summarizing the characteristics of the studies, interventions, comparators, and outcomes. The counts and ranges of each parameter recorded in the data extraction table were analyzed to determine trends across the designs, methodology, and outcomes of the studies. A quantitative meta-analysis was not performed due to considerable heterogeneity among the included studies, particularly in study design (parallel, split-mouth, and multi-arm randomized controlled trials (RCTs)), lesion etiology (orthodontic vs naturally occurring WSLs), outcome measures (DIAGNOdent, QLF, International Caries Detection and Assessment System (ICDAS), Nyvad), and follow-up durations. These variations precluded meaningful statistical pooling and justified the adoption of a narrative synthesis approach in accordance with PRISMA recommendations.

Assessment of Risk of Bias and Quality of Evidence

Methodological quality was assessed using the Cochrane Risk of Bias 2 (ROB‑2) tool [20]. Two independent reviewers evaluated each study across five key domains: the randomization process, deviations from intended interventions, missing outcome data, measurement of the outcome, and the selection of the reported result. For the randomization process, reviewers examined the adequacy of sequence generation and allocation concealment to ensure that participants were assigned to treatment groups without bias. In assessing deviations from intended interventions, particular attention was given to whether blinding was implemented for both participants and operators; however, it was acknowledged that operator blinding was not always feasible due to the nature of the interventions. Missing outcome data were scrutinized by reviewing the completeness of the data and any loss to follow-up, while the measurement of outcomes was judged based on the use of objective, validated instruments (e.g., DIAGNOdent) and whether outcome assessors were blinded. Finally, the selection of the reported result was assessed by comparing published outcomes with pre‑specified protocols to identify potential selective reporting. Any discrepancies or uncertainties were resolved through consensus discussion between reviewers.

The quality of evidence for each outcome was assessed using the Grading of Recommendations, Assessment, Development, and Evaluation (GRADE) approach [21]. The GRADE ratings were assigned based on four core domains including risk of bias, inconsistency, indirectness, and imprecision, following the standard GRADE framework. “High” quality was attributed when further research was very unlikely to change confidence in the estimate of effect; “moderate” when further research might influence the confidence; and “low” when evidence was limited or inconsistent. Disagreements between reviewers were resolved through discussion and consensus, with arbitration by a third reviewer when necessary.

Results

Study Characteristics

**Figure 1:**
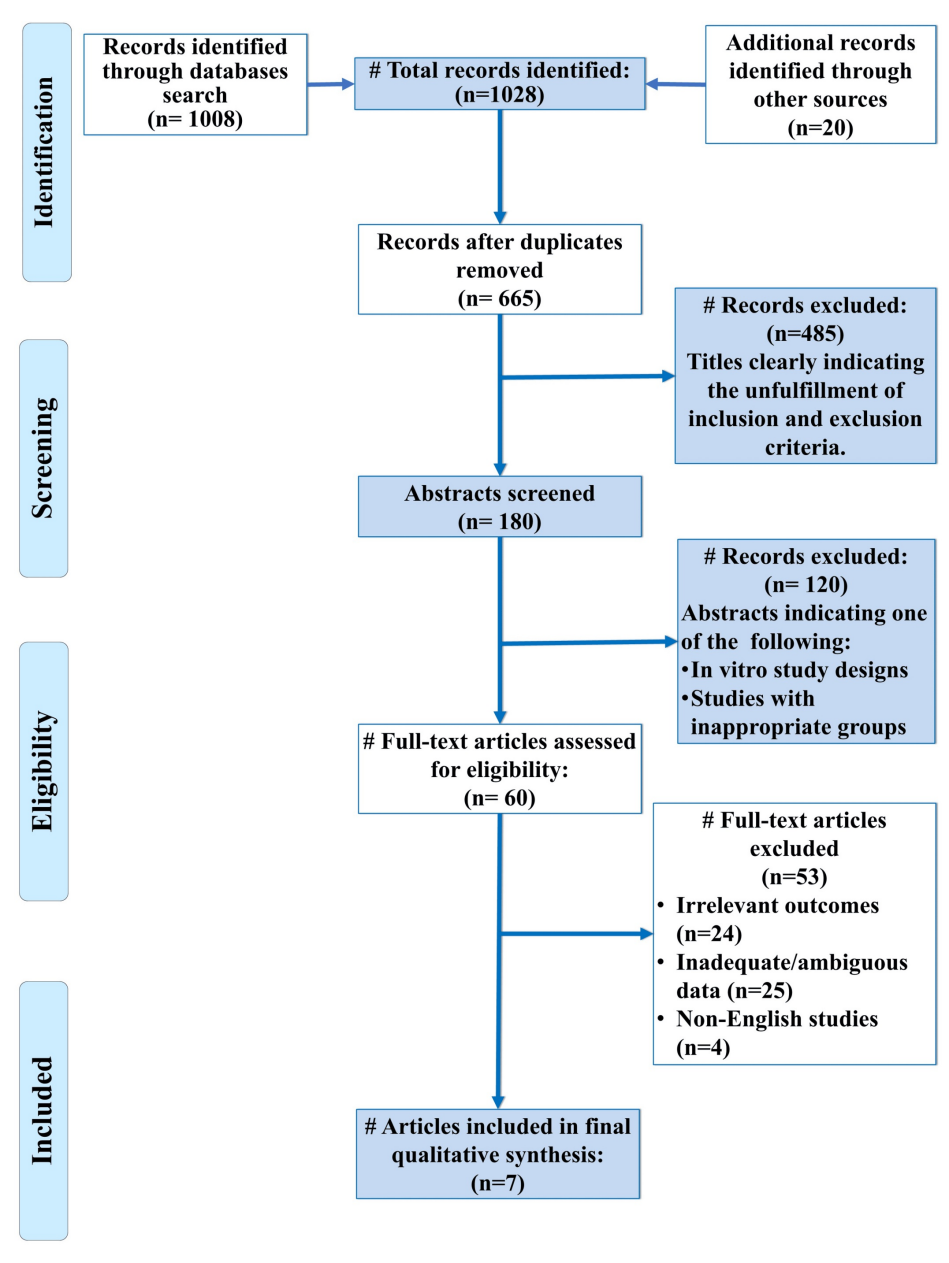
Preferred Reporting Items for Systematic Reviews and Meta-Analyses (PRISMA) Flow Diagram indicating the selection process of the articles in the present systematic review # = number of

A total of 1028 articles were identified through literature search, out of which 665 titles were assessed after removal of duplicates. One hundred eighty abstracts were screened and 60 full texts were assessed for eligiblity. Out of these, seven studies (n=7) were included in this systematic review (Figure 1). Collectively, the included studies encompassed a total of 254 participants and 669 white spot lesions, with participant ages ranging from three to 36 years. This range reflects a diverse population spanning both primary and permanent dentitions, allowing for a broad evaluation of the remineralization efficacy of SAPs and fluoride agents across age groups. The data extracted from these studies is summarized in Table 1. The designs comprised RCTs with split‐mouth, parallel‐group, or double‐blinded methodologies and one prospective in‑vivo study with a four-arm design. The studies were conducted in different geographical settings: one study in Germany, one in Lebanon, and five in Egypt, with one study not specifying its country [22-28]. The earliest study was conducted in 2019, indicating that the interest of researchers in SAP for enamel remineralization is a relatively recent one [22].

**Table 2:** Characteristic data extracted from the included studies SAP: self-assembling peptide; P11-4 (SAP): self-assembling peptide P11-4; CDR: Curodont Repair; WSLs: white spot lesions; NaOCl: sodium hypochlorite; H₃PO₄: phosphoric acid; NaF: sodium fluoride; TCP: tricalcium phosphate; TCPF: tricalcium phosphate fluoride; NSF: nano-silver fluoride; ICDAS II: International Caries Detection and Assessment System II; DIAGNOdent™: quantitative laser fluorescence device; QLF: quantitative light-induced fluorescence; ΔF: percent fluorescence loss; ΔQ: lesion volume metric (fluorescence loss × area); VAS: visual analogue scale; GICQ: Global Impression of Change Questionnaire; AAPD: American Academy of Pediatric Dentistry; SD: standard deviation; RCT: randomized controlled trial.

Sr. No.	Authors & Year	Study Design	Country/Setting	Sample Size (Subjects)	Age Range	Mean Age (SD)	Gender Distribution	Inclusion Criteria	Exclusion Criteria	Type of SAP	SAP Concentration	SAP Application Protocol	Type of Fluoride Agent	Fluoride Concentration	Fluoride Application Protocol	Primary Outcome Measure	Primary Outcome Results	Follow-Up Duration	Risk of Bias / Blinding	Conclusive Findings
1	Bröseler et al., 2019 [22]	Randomised, subject- and assessor-blinded, controlled split-mouth clinical trial	Single dental practice, Aachen, Germany	37 subjects (90 teeth)	13–36 years	21.8 (5.9) years	20 females, 17 males	At least 2 early buccal carious lesions on two teeth not requiring operative intervention; willingness to maintain good oral hygiene	Use of tetracyclines; medications causing tooth staining or dry mouth/limiting salivary flow; enamel anomalies	Self-assembling peptide P11-4 (Curodont® Repair)	Not specified	Pre-treatment with NaOCl cleaning, 35% H₃PO₄ etching for 20 seconds, rinsing; applied on Day 0 and Day 90 to test lesions	Fluoride varnish (Duraphat® Fluoride Varnish)	50 mg/ml	Applied to control lesions at Day 0; all lesions received fluoride varnish on Day 180	Change in lesion size via blinded morphometric analysis (relative to baseline)	Test lesions reduced to 0.862 (SD 0.352) of baseline at Day 360; Control lesions increased to 1.068 (SD 0.401) of baseline	360 days (1 year)	Assessor-blinded for morphometry; subjects blinded; VAS/GICQ assessments by unblinded investigators may introduce bias	P11-4 treatment significantly reduced early carious lesion size compared to fluoride varnish treatment.
2	Kobeissi et al., 2020 [23]	Randomized controlled clinical trial; single-blinded (patient blinded)	Beirut Arab University dental clinics, Beirut, Lebanon	9 subjects (40 teeth)	7–17 years	11.11 (3.8) years	5 girls, 4 boys	Accessible white spot lesions (WSLs) on buccal/labial surfaces of young permanent teeth; DIAGNOdent pen reading <24; ICDAS II codes 1–3; absence of caries, restorations, and cavitations on treated surfaces	Teeth with existing caries, restorations, or cavitations were excluded	Self-assembling peptide P11-4 (Curodont Repair, CDR)	Not specified	Remove pellicle with 5% sodium hypochlorite for 20 sec, rinse and dry; etch with 37% phosphoric acid for 20 sec; rinse for 20 sec and air-dry; apply one drop of SAP11-4 using a special syringe; maintain moisture control for approximately 5 minutes until the solution is no longer visible	Tricalcium phosphate fluoride varnish (Clinpro™ White Varnish)	Not specified	Dispense the entire content of a 1-unit dose package onto the dosage guide, mix with the provided applicator brush, and apply evenly on the tooth surface	Remineralization assessed via decrease in DIAGNOdent pen readings and ICDAS II codes	Both groups showed significant remineralization; SAP11-4 group had significantly greater reduction in DIAGNOdent readings (mean variation percentages: 26.68% at 1 month, 36.11% at 3 months, 41.39% at 6 months) compared to TCPF group (12.97%, 26.51%, 32.72% respectively) along with improved ICDAS II scores	6 months	Single-blinded (patient blinded); random allocation using coin flip with opaque sealed envelopes	Both TCPF and SAP11-4 were effective, but SAP11-4 demonstrated superior remineralization potential due to its guided enamel regeneration capability, despite a more complex application procedure.
3	Atteya et al., 2023 [24]	Parallel randomized controlled clinical trial with three arms	Pediatric Dentistry clinic, Faculty of Dentistry, Alexandria University, Alexandria, Egypt	66 subjects (147 lesions)	10–24 years	13.46 (4.31) years	66.7% females	At least one visible WSL on the buccal surface of permanent teeth; ICDAS II score of 1 or 2; informed consent obtained	Patients receiving tetracyclines or other tooth‐staining medications; fluoride application within 3 months; teeth with microcavities, dentinal involvement, adjacent restorations, discoloration, enamel hypoplasia or fluorosis	Self-assembling peptide P11-4 (Curodont Repair™)	Not specified	Tooth isolated with a rubber dam; cleaned with 2% sodium hypochlorite for 20 s; acid etched with 35–37% phosphoric acid for 20 s; rinsed and dried; P11-4 applied by activating the applicator and squeezing out the sponge; left to diffuse for 5 min	Nano-silver fluoride varnish (NSF) and 5% sodium fluoride varnish (NaF, Duraflor®)	NSF: Not specified; NaF: 5%	NSF: Two drops applied at baseline, left in contact for 2 min; NaF: Thin coat applied at baseline and after 6 months, allowed to dry for 10 s	Changes in ICDAS II scores and NYVAD lesion activity scores	At 12 months, ICDAS score reduction was 54.5% in the P11-4 group, 47.7% in the NSF group, and 30.5% in the NaF group; lesion activity showed 100% inactive lesions in the P11-4 group versus 81.4% in NSF and NaF; Diagnodent readings were lowest in the P11-4 group; however, adjusted analysis revealed no statistically significant differences in ICDAS reduction among groups	12 months	Participants and outcome assessor blinded; the examiner administering the intervention was not blinded	P11-4 and NSF demonstrated greater remineralizing effects (lower ICDAS scores, reduced lesion activity, and lower Diagnodent readings) compared to NaF, though adjusted differences were not statistically significant.
4	Gohar et al., 2023 [25]	Randomized controlled trial with two parallel groups (1:1 allocation)	Department of Conservative Dentistry, Cairo University, Egypt	58 subjects (29 per group)	18–25 years	Group A: 21.62 ± 2.78; Group B: 21.45 ± 2.53 (~21.5 ± 2.65 years)	Group A: 14 males (48.3%), 15 females (51.7%); Group B: 11 males (37.9%), 18 females (62.1%)	Patients aged 18–25 years with active, non-cavitated post‑orthodontic white spot lesions; good oral hygiene (plaque index 0–1); completed fixed orthodontic treatment within the past 2 weeks; good general health and compliance	Patients with tetracycline pigmentation, dental fluorosis, enamel hypoplasia; reduced salivary flow; cavitated lesions; or participation in another trial	Self-assembling peptide P11‑4 (Curodont repair™, Credentis AG, Windisch, Switzerland)	Not specified	Pre-treatment: Clean tooth with 2% NaOCl for 20 s, rinse for 20 s, air-dry; etch with 35% phosphoric acid for 20 s, rinse for 20 s; then apply Curodont repair™ using an activated applicator and leave for 5 min until the tooth surface appears dry	Fluoride varnish containing 5% sodium fluoride and tricalcium phosphate (Clinpro White Varnish, 3M ESPE)	5% sodium fluoride	Apply a thin layer with a brush (drying not required); no rinsing/suction; instruct patient to avoid brushing, flossing, and solid foods for 4 h post-application	Changes in remineralization measured quantitatively by DIAGNOdent readings and qualitatively by ICDAS scores at baseline, 3 and 6 months	Quantitative (DIAGNOdent): Group A (fluoride) showed a highest mean value of 10.51 and Group B (SAP) the lowest mean value of 6.45 at 6 months (p < 0.001); Qualitative (ICDAS): No significant differences between groups at T0, T1, and T2 (p = 0.064, 0.087, 0.277, respectively)	6 months	Patients and outcome assessor were blinded; the operator was not blinded due to differences in material presentation and application protocol	Self-assembling peptide P11‑4 demonstrated superior subsurface remineralization (as evidenced by significantly lower DIAGNOdent readings) compared to fluoride varnish, although both materials produced similar visual masking of lesions (ICDAS scores).
5	Mamdouh et al., 2023 [26]	Double-blinded randomized controlled clinical trial	Pediatric Dentistry and Dental Public Health Department, Faculty of Dentistry, Alexandria University, Egypt	24 children	3–6 years	Study group: 4.79 ± 0.89; Control group: 5.00 ± 0.85	Not explicitly reported	Healthy children at high caries risk with at least one visible active white spot lesion on primary teeth (ICDAS II score 1–3; active per Nyvad criteria)	Not explicitly detailed (only children not meeting inclusion criteria were excluded)	Self-assembling peptide P11‑4 (Curodont™ Repair)	Not specified	Under rubber dam isolation, the tooth surface was cleaned with 2% NaOCl for 20 s, then etched with 35–37% phosphoric acid for 20 s, rinsed and dried; SAP (P11‑4) was applied per manufacturer’s instructions and left to diffuse for 5 min until dry, followed by application of a thin coat of 5% NaF varnish (using a brush, allowed to dry for 10 s)	5% Sodium Fluoride varnish (Dura�or)	5%	Applied as a thin coat with a brush; re-applied at 3 and 6 months per AAPD guidelines	Change in caries status of WSLs as measured primarily by DIAGNOdent™ readings (quantitative laser fluorescence), with secondary assessment by ICDAS II and Nyvad criteria	In the test group (SAP + 5% NaF varnish), DIAGNOdent™ readings decreased significantly from baseline to 6 months (median reduction from 14.00 to 10.50; p=0.009) with percent reductions of −23.76% (baseline to 3 months, p=0.026) and −31.48% (baseline to 6 months, p=0.020); ICDAS II scores showed no significant change; at 6 months, 75% of lesions in the test group regressed versus 58.3% in the control group (difference not statistically significant for lesion regression, p=0.676)	6 months	Double-blinded: both patients and outcome assessor were blinded; the operator was not blinded due to differing treatment protocols	SAP (P11‑4) in conjunction with 5% NaF varnish demonstrated superior remineralization—evidenced by a significantly greater reduction in DIAGNOdent™ readings over 6 months—compared to 5% NaF varnish alone.
6	Shaalan et al., 2024 [27]	Randomized controlled clinical trial (parallel design, 1:1 allocation)	Cairo University, Egypt	28 participants (58 lesions)	Not specified	Not specified	Not specified	Participants with non-cavitated incipient carious lesions	Not specified	Self-assembling peptide P11‑4 (Curodont Repair Fluoride Plus™)	Not specified	Applied according to the manufacturer’s instructions on non-cavitated incipient lesions	Sodium fluoride varnish (Bifluorid 10)	5% (assumed)	Applied per manufacturer’s instructions on non-cavitated incipient lesions	Laser fluorescence readings (DIAGNOdent)	At 3 and 6 months, DIAGNOdent readings significantly improved in both groups, with the SAP+fluoride group showing statistically lower readings (p < 0.05); 60% less risk for caries progression; 65.5% of lesions in the SAP+fluoride group shifted from score 3 (11–20) to score 1 (0–4) versus 13.8% in the NaF group	6 months	Outcome assessors were blinded	The combination of self-assembling peptide P11‑4 with fluoride demonstrated superior remineralization potential compared to fluoride varnish alone over a six‑month follow‑up period.
7	Güven et al., 2024 [28]	Prospective in‑vivo study with four groups (control, TCP fluoride varnish, SAP, combined SAP+fluoride)	Not specified	32 subjects (107 lesions)	10–18 years	13.91 ± 2.92 years	Not specified	Post‑orthodontic non‑cavitated incipient WSLs on teeth	Not specified	Self‑assembling peptide P11‑4 (Curodont Repair) used alone and in combined form (Curodont Repair Fluoride Plus™)	Not specified	Applied per manufacturer’s instructions on non‑cavitated lesions	Sodium fluoride varnish with TCP component (Clinpro White varnish)	5%	Applied per manufacturer’s instructions; for combined group, SAP and fluoride applied concurrently	Remineralization assessed by QLF (ΔF, ΔQ, lesion area) and DIAGNOdent	At 6 months, inter‑group comparisons showed highest remineralization with fluoride varnish with TCP; SAP and combined groups showed significant short‑term improvement at 3 months; combined group exhibited 60% less risk for caries progression and 65.5% of lesions shifted from DIAGNOdent score 3 to 1 versus 13.8% in the fluoride group	6 months	Outcome assessors blinded; randomization details not fully specified	Fluoride varnish with TCP achieved highest remineralization at 6 months; SAP (alone and combined) improved short‑term remineralization; combined use warrants further evaluation for synergistic effects.

Population and Demographics

The review included diverse populations ranging from preschool children to young adults [22-28]. In studies conducted in children and adolescents, sample sizes ranged from nine subjects (Kobeissi et al., 2020; ages seven to 17 years) and 24 children (Mamdouh et al., 2023; ages three to six years) to 32 subjects (Güven et al., 2024; ages 10 to 18 years). In young adult populations, Bröseler et al. (2019) enrolled 37 subjects with ages spanning 13 to 36 years (mean 21.8 ± 5.9 years), and Gohar et al. (2023) studied 58 subjects (29 per group) aged 18 to 25 years. Atteya et al. (2023) included 66 subjects (ages 10 to 24 years), representing a mix of adolescents and young adults. Shaalan et al. (2024) investigated incipient carious lesions in 28 participants, although the specific age range was not provided.

Interventions and Comparator Treatments

All studies evaluated the remineralization potential of self‑assembling peptide P11‑4, commercially available as Curodont Repair (Credentis AG, Windisch, Switzerland) or its modified formulations. The application protocol across studies was similar: the lesion area was first cleaned using a sodium hypochlorite solution (ranging from 2% to 5% for approximately 20 seconds) followed by acid etching with phosphoric acid (35-37% for 20 seconds) to expose the subsurface lesion. The SAP solution was then applied according to manufacturer instructions and left in place for approximately five minutes to allow the formation of a three‑dimensional matrix [22-28].

The comparators in these studies were various fluoride-based agents. Bröseler et al. (2019) used Duraphat® Fluoride Varnish (50 mg/ml; Colgate-Palmolive GmbH, Hamburg, Germany), while Kobeissi et al. (2020) and Gohar et al. (2023) employed Clinpro™ White Varnish (3M ESPE Dental Products, St. Paul, MN, USA), which includes tricalcium phosphate combined with 5% sodium fluoride [23,25]. Shaalan et al. (2024) utilized Bifluorid10 (VOCO GmbH, Cuxhaven, Germany) as the fluoride varnish, and Atteya et al. (2023) incorporated nano‑silver fluoride varnish alongside SAP P11‑4 and a fluoride-alone arm [24,27]. Mamdouh et al. (2023) compared a combination treatment (SAP P11‑4 with 5% NaF varnish) to fluoride varnish alone [26]. Güven et al. (2024) evaluated four arms: a control group, a group receiving tricalcium phosphate (TCP)-containing fluoride varnish, a group receiving SAP P11‑4 alone, and a combined application group [28].

Outcome Measures

The primary outcome measure in all studies was the degree of remineralization, predominantly assessed quantitatively using laser fluorescence devices such as DIAGNOdent (KaVo Dental GmbH, Biberach, Germany). DIAGNOdent readings served as an indicator of subsurface mineral content, where a reduction from baseline reflected remineralization. For example, Kobeissi et al. (2020) reported mean DIAGNOdent reading reductions corresponding to approximately 26.7% at one month, 36.1% at three months, and 41.4% at six months [23]. Gohar et al. (2023) observed significantly lower DIAGNOdent values in the SAP group (mean 6.45) compared to the fluoride group (mean 10.51) at six months (p < 0.001) [25]. Mamdouh et al. (2023) documented a significant DIAGNOdent reduction (p=0.009) with 75% lesion regression in the SAP+NaF group compared to 58.3% in the control group [26]. Shaalan et al. (2024) further demonstrated that the SAP+fluoride group had a 60% lower risk for caries progression and that 65.5% of lesions shifted from a DIAGNOdent score of 3 to 1 versus only 13.8% in the fluoride varnish group [27].

Secondary outcome measures included qualitative assessments of lesion appearance using the ICDAS and lesion activity evaluation using the Nyvad criteria [29]. Several studies noted that although DIAGNOdent readings improved significantly in the intervention groups, the ICDAS scores did not differ significantly between the groups over time, suggesting that enhanced subsurface remineralization might not always translate into markedly different surface appearances [30].

Follow‑Up Duration and Blinding

Follow‑up durations varied among the studies. Five studies used a six‑month follow‑up period, whereas Atteya et al. (2023) extended the follow‑up to 12 months [22-27]. In terms of blinding, outcome assessors were blinded in all studies. In most trials, patients were also blinded, although operator blinding was often not feasible due to distinct application protocols [23-25,27].

Quantitative Outcomes

Quantitative outcomes, as measured by DIAGNOdent, consistently demonstrated significant reductions in fluorescence values in the groups receiving SAP P11‑4. The degree of improvement ranged from a reduction of approximately 23% up to over 41% from baseline, with studies such as Kobeissi et al. (2020) and Gohar et al. (2023) reporting statistically significant differences in DIAGNOdent values favoring the SAP groups over the fluoride-only groups [23,25]. Additionally, Mamdouh et al. (2023) and Shaalan et al. (2024) provided evidence that combining SAP with fluoride varnish resulted in greater remineralization, as indicated by lower DIAGNOdent readings and higher percentages of lesion regression [26,27].

Qualitative Outcomes

Qualitative assessments using ICDAS and Nyvad criteria were performed in several studies. Despite the marked improvements in DIAGNOdent readings, ICDAS scores frequently remained similar between intervention and control groups over time. This observation suggests that while subsurface remineralization is enhanced by SAP P11‑4, the changes may not be as easily discernible through visual inspection alone. For instance, Gohar et al. (2023) and Mamdouh et al. (2023) found no statistically significant differences in ICDAS scores between the groups, highlighting a potential disconnect between quantitative subsurface changes and surface visual appearance [25,26].

O*verall Synthesis*

The narrative synthesis indicates that SAP P11‑4, whether used alone or in combination with fluoride varnish, consistently exhibits superior subsurface remineralization potential. DIAGNOdent data across studies revealed reductions ranging from 23% to 41%, with studies in pediatric populations (Mamdouh et al. and Shaalan et al.) showing that the combination of SAP with 5% NaF varnish significantly reduced DIAGNOdent values and minimized the risk for caries progression. Beyond statistical significance, several studies reported that these quantitative reductions translated into clinically meaningful outcomes such as lesion inactivation, reduced caries progression, and improved surface hardness, suggesting true clinical relevance of the observed remineralization. Although visual assessments (ICDAS) did not show significant differences, the quantitative improvements support the enhanced mineral deposition in the subsurface, potentially offering a minimally invasive therapeutic option for early carious lesions.

Overall, the data from the seven studies (n=7) indicate that self‑assembling peptide P11‑4-based interventions provide a notable improvement in remineralization compared to fluoride varnish alone [22-28]. The overall trend across studies demonstrates that SAP P11‑4, particularly when combined with fluoride, results in greater subsurface mineral gains as evidenced by DIAGNOdent readings, even though these changes are not always evident in the surface appearance of lesions. Further long‑term research is recommended to standardize protocols and validate the clinical benefits of these biomimetic interventions.

Risk of Bias Assessment

**Figure 2:**
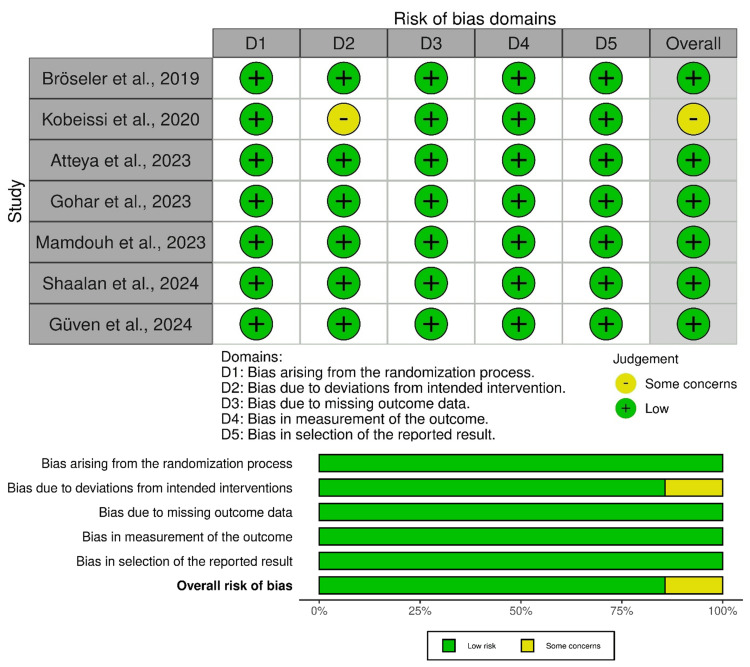
Risk of bias across the included studies [22-28]

Overall, the risk of bias assessment using the Cochrane ROB‑2 tool indicates that six out of the seven studies were judged to have a low risk of bias across all domains (Figure 2). Bröseler et al. (2019), Atteya et al. (2023), Gohar et al. (2023), Mamdouh et al. (2023), Shaalan et al. (2024), and Güven et al. (2024) all demonstrated adequate randomization processes, blinding of outcome assessors, minimal missing outcome data, and low risk of selective reporting. Kobeissi et al. (2020) was noted to have some concerns in the domain of deviations from intended interventions due to the inability to blind the operator; however, the objective nature of the outcome measurements (e.g., DIAGNOdent readings) mitigates this concern. Overall, the methodological quality across the studies is high, supporting the internal validity of the findings reported in this systematic review. Publication bias was not formally evaluated using funnel plots due to the limited number of studies (n=7); however, a qualitative assessment indicated a predominance of small, single-center trials reporting positive outcomes, suggesting a possible overrepresentation of favorable findings in the available literature.

Quality of Evidence

Overall, the evidence quality ranged from low to high across the studies. Bröseler et al. (2019) was assigned a moderate quality rating [22]. Although this study demonstrated a low risk of bias, directness, and consistency, its overall quality was limited by moderate imprecision, suggesting that while the findings are promising, the confidence in the effect estimate is not concrete enough to rule out a clinically meaningful difference. In contrast, Kobeissi et al. (2020) received an overall low-quality rating. Despite having a low risk of bias in the randomization process and measurement of outcomes, concerns related to deviations from intended interventions (owing to the lack of operator blinding), moderate inconsistency, and high imprecision reduced our confidence in the reliability of its results [23].

On the other hand, Atteya et al. (2023), Gohar et al. (2023), Mamdouh et al. (2023), and Shaalan et al. (2024) were all rated as high-quality evidence [23,25-27]. These studies consistently demonstrated low risk of bias, minimal inconsistency and indirectness, and low imprecision. Their good quality of methodology and comprehensive reporting provide high confidence in their effect estimates. Güven et al. (2024) was judged as moderate quality overall due to moderate inconsistency and imprecision, despite a low risk of bias and indirectness [28].

The GRADE assessment (Table 3) indicates that the majority of the evidence supporting the efficacy of self‑assembling peptide P11‑4 (alone or combined with fluoride varnish) for remineralization of white spot lesions is of high quality, particularly in four studies [24-27]. However, some caution is warranted when interpreting findings from Kobeissi et al. (2020) due to concerns regarding imprecision and potential performance bias. These results reinforce the overall conclusion of this review that SAP P11‑4‐based interventions are effective in enhancing subsurface remineralization, although differences in study design and outcome measurement methods contribute to some variability in the evidence quality.

**Table 3:** Assessment of quality of evidence using Grading of Recommendations, Assessment, Development, and Evaluation (GRADE) approach

Study	Risk of Bias	Inconsistency	Indirectness	Imprecision	Publication Bias	Overall Quality of Evidence
Bröseler et al., 2019 [22]	Low	Low	Low	Moderate	Unclear	Moderate
Kobeissi et al., 2020 [23]	Some concerns	Moderate	Low	High	Unclear	Low
Atteya et al., 2023 [24]	Low	Low	Low	Low	Unclear	High
Gohar et al., 2023 [25]	Low	Low	Low	Low	Unclear	High
Mamdouh et al., 2023 [26]	Low	Low	Low	Low	Unclear	High
Shaalan et al., 2024 [27]	Low	Low	Low	Low	Unclear	High
Güven et al., 2024 [28]	Low	Moderate	Low	Moderate	Unclear	Moderate

Discussion

This systematic review highlights the potential of self-assembling peptide P11-4 as an effective, minimally invasive agent for remineralizing WSLs in both primary and permanent dentition. The included studies consistently demonstrated reductions in DIAGNOdent values following treatment with SAP P11-4, whether used alone or in combination with fluoride varnish. These outcomes support the hypothesis that SAPs, by replicating the physiological mineralization process, can regenerate enamel structure through controlled hydroxyapatite deposition [31]. SAP P11-4 promotes subsurface mineral regeneration by forming a biomimetic scaffold within demineralized enamel, enabling deeper and more uniform hydroxyapatite deposition than the surface-limited effect typically achieved with fluoride [10,32].

When compared directly, while fluoride varnish was effective in inhibiting demineralization, it did not consistently match the restorative potential of SAP P11-4 due to its restricted penetration and limited capacity to rebuild deeper enamel structure [33,34]. This was evident in studies such as those by Gohar et al. (2023) and Kobeissi et al. (2020), where SAP P11-4 groups achieved greater reductions in DIAGNOdent readings, indicating enhanced mineral deposition at the lesion core [23,25]. These findings are particularly relevant considering that WSLs originate beneath a seemingly intact surface layer, underscoring the clinical importance of a remineralization approach that targets internal enamel porosity [4,31].

Interestingly, although DIAGNOdent values improved significantly, the same degree of improvement was not consistently reflected in ICDAS scores. Studies such as those by Gohar et al. (2023) and Mamdouh et al. (2023) reported minimal differences in visual lesion assessments between groups [25,26]. This discrepancy can be attributed to the optical limitations of early remineralized enamel. Due to mismatched refractive indices between new mineral deposits and native enamel, lesions may continue to appear opaque despite substantial subsurface recovery [35,36]. Studies on enamel translucency recovery have confirmed that even when mineral content approaches normal values, incomplete realignment of enamel prism orientation and residual porosity can delay optical normalization [37,38]. Over time, maturation of newly formed hydroxyapatite and reorganization of prism structures may gradually restore translucency, although the process is non-linear and varies with lesion depth and mineral density. This further underscores that visual indices such as ICDAS may be inherently less sensitive to early mineral changes than fluorescence-based methods like DIAGNOdent or QLF, which can detect subsurface alterations undetectable to the naked eye. Consequently, quantitative fluorescence assessments provide a more precise measure of lesion regression in the early phases of remineralization. Therefore, long-term follow-ups are essential to determine whether mineral maturation and crystal reorganization eventually reduce visual opacity.

The synergistic potential of SAP P11-4 when combined with fluoride varnish was another key finding. Studies including those by Mamdouh et al. (2023) and Shaalan et al. (2024), showed that the combination treatment yielded greater remineralization benefits than fluoride alone [10,22-28]. This enhanced efficacy likely results from the complementary actions of SAP and fluoride, where SAP builds the internal scaffold and fluoride stabilizes and strengthens the outer enamel [39]. Shaalan et al. (2024) also reported a 60% reduction in caries progression risk, reinforcing the clinical promise of this dual approach [27].

Variation in lesion etiology and depth also influenced treatment response. SAP P11-4 was effective in both orthodontic-induced and naturally occurring WSLs, as seen in the study by Atteya et al. (2023) [24]. However, lesion depth likely affects the pace and extent of remineralization, with superficial lesions responding faster. Moreover, studies with longer follow-up, such as Atteya et al. (2023), confirmed that mineral gains from SAP use are durable over time, which is critical for long-term lesion stability and relapse prevention [24].

While the overall results favor SAP P11-4, limitations such as heterogeneity in fluoride comparators and absence of operator blinding must be acknowledged [22-28]. The restriction to English-language publications may have introduced a potential language bias, as relevant studies published in other languages were excluded. Additionally, although grey literature sources such as conference abstracts and dissertations were included to minimize publication bias, the variable methodological quality and limited data from such sources could have influenced the overall strength of the evidence. These factors should be considered when interpreting the findings and generalizability of this review.

Moreover, variability in lesion etiology, such as that between orthodontic-induced and naturally occurring WSLs, may have influenced treatment response and contributed to inter-study heterogeneity. Future clinical trials should control for this variable through stratified randomization and employ standardized, calibrated outcome measures such as DIAGNOdent thresholds, ICDAS scoring, and quantitative light-induced fluorescence metrics to ensure consistency and comparability across studies. Additionally, reliance on DIAGNOdent alone may restrict interpretative depth. Despite the comprehensive inclusion and exclusion criteria applied in this review, certain methodological limitations must be acknowledged. Future studies should employ imaging tools like micro-CT to map mineral recovery more precisely, and standardized fluoride protocols should be adopted to facilitate more valid comparisons.

## Conclusions

Within the limits of the available short-term clinical trials, the findings of this systematic review establish SAP P11-4 as a promising non-invasive intervention for white spot lesion management, demonstrating superior subsurface remineralization compared to fluoride-based treatments. The ability of SAP to penetrate and mineralize lesions at deeper levels distinguishes it from conventional fluoride therapies, offering an innovative approach to early caries intervention. The evidence further suggests that the combination of SAP and fluoride may enhance remineralization outcomes, presenting an opportunity for optimized treatment protocols. Nevertheless, these conclusions should be interpreted with caution, and well-designed, long-term randomized controlled trials are required to validate these findings, refine application protocols, and determine the durability of enamel regeneration. These insights add to the growing body of literature supporting biomimetic strategies in modern dentistry, underscoring the potential of SAP as a breakthrough in minimally invasive caries management.

## Figures and Tables

**Figure 3:**
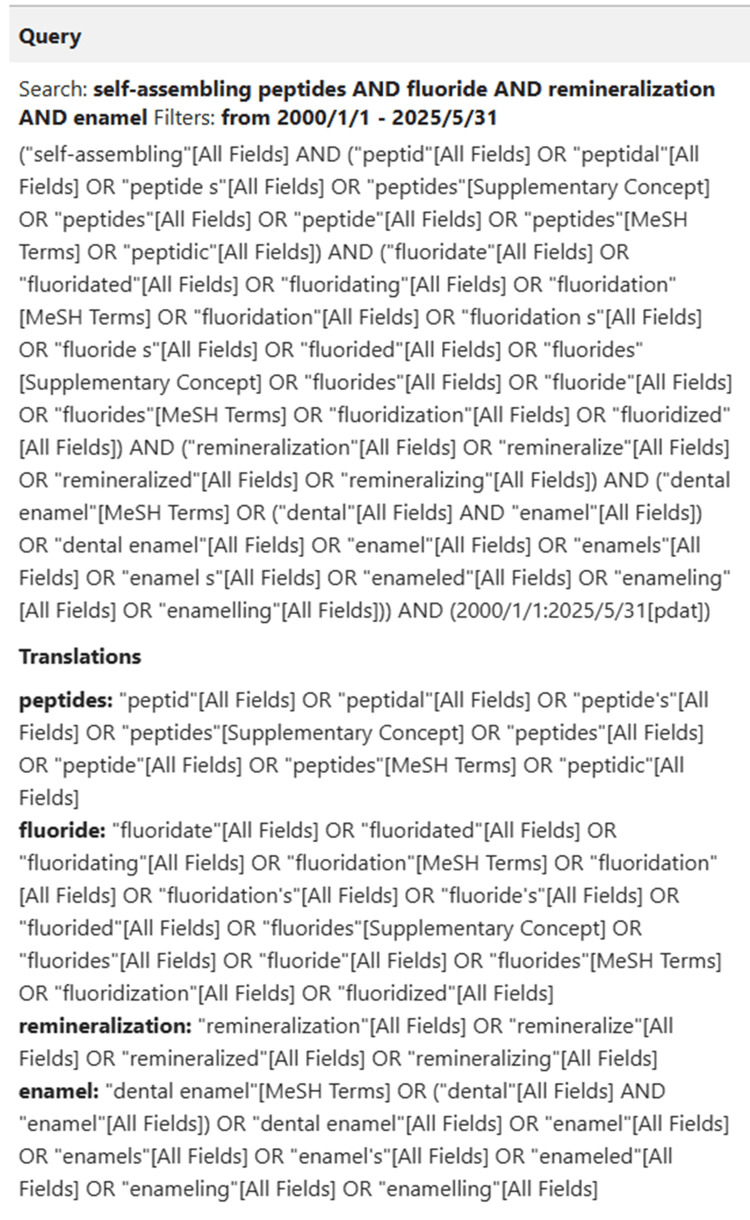
Sample search string in PubMed
